# Elevated Preoperative NMPR Predicts an Unfavorable Chance of Survival in Resectable Esophageal Squamous Cell Carcinoma

**DOI:** 10.3390/medicina58121808

**Published:** 2022-12-08

**Authors:** Meng-Ying Peng, Zhi-Gang Zuo, Feng-Jun Cao, Yuan-Dong Yu, Xiao-Jun Cai, Guo-Xing Wan

**Affiliations:** 1 Graduate Student Training Base, Graduate School of Jinzhou Medical University, Jinzhou 121004, China; 2 Department of Oncology, Renmin Hospital, Hubei University of Medicine, Shiyan 442000, China; 3 Institute of Cancer, Renmin Hospital, Hubei University of Medicine, Shiyan 442000, China

**Keywords:** esophageal squamous cell carcinoma, neutrophil–mean-platelet-volume–platelet ratio, prognosis, biomarker

## Abstract

*Background and objectives*: Combined peripheral neutrophil–platelet indexes reflecting the systemic inflammatory status have been reported to predict the clinical outcome in patients with various types of cancer. However, the prognostic value of combined neutrophil–platelet indexes in operable esophageal squamous cell carcinoma (ESCC) remains unclear. The study introduced a novel combined neutrophil–meanplateletvolume–platelet ratio (NMPR) index and investigated its clinical and prognostic value in patients with operable ESCC receiving curative surgery. *Materials and Methods*: A retrospective analysis of the clinicopathologic data of 277 consecutive ESCC patients who received curative resection at Zhejiang Cancer Hospital in China between January 2007 and December 2010 was conducted (the training cohort). In addition, the clinicopathologic data of 101 resectable ESCC patients at Renmin Hospital of Hubei University of Medicine between December 2018 and June 2021 were collected (the external validation cohort). The optimal cutoff value of NMPR concerning overall survival (OS) in the training cohort was determined by X-tile software. Univariate and multivariate Cox regression analyses were used to evaluate the prognostic value of NMPR along with other variables in the training cohort, which was further validated with the same cutoff value in the external validation cohort. Significant predictors of OS were used to construct the nomogram, of which the discrimination and calibration was evaluated by concordance index (C-index) and calibration plots. *Results*: With a cutoff value of 16.62, the results from both the training and external validation cohorts supported the association of high NMPR (>16.62) with increased tumor length and advanced T stage but not with other variables. In the training cohort, a significant association between shorter OS and high NMPR (*p* = 0.04) as well as high CRP (*p* < 0.001), poor tumor differentiation (*p* = 0.008), advanced T stage (*p* = 0.006), advanced N stage (*p* < 0.001) and high CEA (*p* = 0.007) was revealed. Additionally, the high NMPR was verified to independently predict unfavorable OS (*p* = 0.049) in the external validation cohort. The C-index of the OS nomogram cooperating significant predictors in the training cohort was 0.71 and the calibration plots of the OS nomogram fitted well. *Conclusions*: The present study demonstrates that high NMPR is an independent predictor of unfavorable OS in resectable ESCC patients without neoadjuvant therapy.

## 1. Introduction

Esophageal cancer (EC) ranks sixth in annual incidence and fourth in mortality in China [1]. Unlike in Western countries, esophageal squamous cell carcinoma (ESCC) is the predominant histopathological type accounting for approximately 90% of the EC cases in Asian populations [2]. Surgical resection with or without chemoradiotherapy remains the mainstay of curative treatment for operable ESCC [3]. Although great progress in perioperative techniques, staging methods, surgical and oncological management has been made, the prognosis of operable ESCC patients remains poor due to tumor recurrence [4]. Some clinicopathologic characteristics including performance status, nutrition status, tumor location, tumor differentiation, vessel invasion, TNM (tumor, node, metastasis) stage, extent of surgical resection, and response to chemoradiotherapy are firmly considered prognostic in operable ESCC [5,6,7]. However, disease recurrence or progression in ESCC patients is not solely determined by the clinicopathologic characteristics of the tumor, and host-related factors including the systemic inflammatory response may have a significant impact as well [8]. Therefore, accurate identification of prognostic factors is critical for the comprehensive evaluation of clinical outcomes and optimization of the treatment strategies.

Accumulating evidence demonstrates that systemic inflammatory status reflected by some peripheral blood parameters such as neutrophils, lymphocytes and platelets, play vital roles in the progression of various cancers [9]. Particularly, neutrophils and platelets, as important pro-tumor inflammatory cells in circulating system, can release many cytokines, chemokines and growth factors, and thus lead to tumor growth and metastasis [10]. Indeed, a variety of inflammatory indicators, such as the neutrophil-to-lymphocyte ratio (NLR), platelet-to-lymphocyte ratio (PLR) and mean-platelet-volume-to-platelet-count ratio (MPV/PC) have been identified as prognostic biomarkers in cancers, including ESCC [3,11,12]. Moreover, a recent study reported the improved ability of preoperative combined NLR and PLR score (CNPS) compared to NLR or PLR alone in predicting postoperative survival in early-stage gastric cancer [13]. Additionally, the CNPS has been found to independently predict the overall survival (OS) in ESCC patients treated with chemoradiotherapy [14,15]. More recently, the combined neutrophil–platelet score (NPS) was found to predict survival in a variety of common solid cancers, including gastroesophageal cancer [16]. However, the prognostic value of the combined neutrophil–platelet index in resectable ESCC patients remains to be elucidated. Furthermore, the previously defined neutrophil–platelet indexes only include the number of platelets while the increased release of biological active factors in a systemic inflammatory condition is mainly derived from the activated platelets, and the findings were not externally validated. We suppose that taking the neutrophil, platelet and MPV, which is recognized as an indicator of platelet activation, into account together may be a more viable means of constructing the neutrophil–platelet index for survival prediction.

Therefore, we introduce a novel combined neutrophil–MPV–platelet ratio (NMPR) index in the present study and evaluate the prognostic value of NMPR in resectable ESCC patients.

## 2. Materials and Methods

### 2.1. Study Population and Design

The study population was comprised of patients from the training cohort (ZJ cohort) and the external validation cohort (RM cohort) in China. The raw data of the training cohort was derived from the public dataset from a retrospective study conducted by Feng et al. [17], which could be obtained without further permission and ethic approval according to the public policy and ethic statement of the dataset. In the training cohort, a total of 277 resectable ESCC patients were recruited between January 2007 and December 2010 at Zhejiang Cancer Hospital. Another 101 patients with ESCC receiving radical resection at Renmin Hospital of Hubei University of Medicine from December 2018 and June 2021 were enrolled as the external validation cohort. All ESCC patients were pathologically confirmed and the data of blood examination were obtained preoperatively. The design and patient exclusion criteria have been previously reported [17]. Briefly, eligible patients were required with complete pretreatment laboratory data of interest and complete follow-up data, including OS or progression-free survival (PFS), but without preoperative treatment, any form of acute inflammatory diseases or infections, unstable or uncontrolled systemic diseases and perioperative distant metastases. The study was designed to evaluate the prognostic value of NMPR in the training cohort and verify it in the external validation cohort. Approval for the study was granted by the Ethics Committee of Renmin Hospital of Hubei University of Medicine with written informed consent from all participants.

### 2.2. Data Collection and Definition

Clinicopathologic data were collected, including age, gender, tumor location (upper, middle and lower), tumor differentiation (well, moderate and poor), tumor length, vessel invasion and tumor stage. Laboratory data including neutrophil count (10^9^/L), mean platelet volume (MPV, fL), platelet count (PC, 10^9^/L), C-reaction protein (CRP, mg/L) and carcinoembryonic antigen (CEA, ng/mL) levels were collected from the blood test results within one week prior to surgery. Furthermore, the survival data in the training cohort (ZJ cohort) were obtained from the dataset provided by Feng et al. [17]. The OS and PFS survival data in the external validation cohort (RM cohort) were obtained according to the follow-up as follows: once every 3–6 months for years 1–2, once every 6 months for years 3–5, and then once every year after year 5 with the assessment of physical examination, thoracic and abdominal computed tomography (CT), routine blood tests, hepatic and renal function tests, and blood tumor marker tests. In our laboratory (RM cohort), blood routine test was performed through automated blood cell counter (Sysmex XN-90000; Sysmex, Kobe, Japan), while CRP level was analyzed with a Hitachi Modular P800 (Hitachi High-tech Corporation, Tokyo, Japan) and CEA level was measured using a Cobas e 601 auto-analyzer (Roche Diagnostics International Ltd, Rotkreuz, Switzerland). In the ZJ cohort, the blood routine test, CRP and CEA levels were measured as previously described [17]. All tumor stage was determined according to the American Joint Committee on Cancer (AJCC-7) classification system and the histopathologic postoperative pTNM stage (tumor-node-metastasis) categories system (International Union against Cancer; UICC-7). NMPR was calculated as follows: NMPR = neutrophil × MPV/log10(PC).

### 2.3. Statistical Analyses

The optimal cutoff level for NMPR to predict OS in training cohort was determined using the X-tile software (version:3.6.1, Copyright Yale University 2003–2005), and this cutoff value was used to divide the study population into high-NMPR and low-NMPR groups. The differences of clinicopathologic parameters between groups were analyzed by the Chi-square test. Time-dependent receiver operating characteristics (ROC) curve was used to evaluate the prediction ability of NMPR, which indicated by the areas under the ROC curve (AUROC) value. The survival curve as well as cumulative survival rates and median OS and PFS was plotted and calculated by the Kaplan–Meier method. Significant prognostic parameters in univariate Cox regression analysis were selected for multivariate Cox regression analysis to identify independent prognostic factors and the corresponding hazard ratio (HR) and 95% confidence intervals (95% CIs) were obtained. All the statistical analyses were performed using SPSS 26.0 statistics software (IBM, Chicago, Illinois) and R-based packages (survival [version 3.4.0], survminer [version 0.4.9], survivalROC [version 1.0.3], pROC [version 1.18.0]). Moreover, the nomogram was constructed with the independent prognostic factors of OS in the training cohort and established idiomatically by the R-based rms package (version 6.3.0) according to the instruction. The performance of the nomogram was evaluated by Harrell’s concordance index (C-index) and calibration plot. The methods for nomogram construction and evaluation were detailed as our previous study [18]. For all statistical analyses, a 2-side *p* < 0.05 was considered statistically significant.

## 3. Results

### 3.1. Clinicopathologic Characteristics

A total of 277 patients with resectable ESCC were incorporated into the present study as the training cohort and 101 resectable ESCC patients were enrolled as the external validation cohort (Table 1). The median follow-up time was 46.1 and 24.5 months for the test and validation cohort, respectively. The median age was 59 (36–80) in the training cohort and 62 (42–78) in the external validation cohort. Some baseline characteristics between the test and validation cohort were remarkably different except for gender, tumor differentiation and T stage (Table 1). More patients with age >60 years, tumor length <3.0 cm, lower location of tumor, N0 stage and earlier TNM stage were found in the validation cohort than that in test cohort (Table 1). Moreover, the preoperative CEA and CRP levels were not comparable due to the lack of relevant information in the validation cohort.

### 3.2. Associations between NMPR and Clinicopathologic Parameters

**Figure 1:**
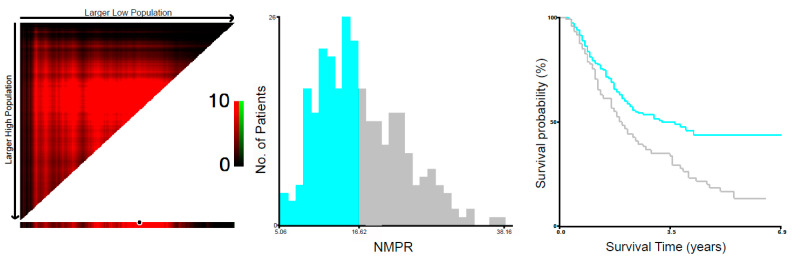
Determination of cut-off values of NMPR level in the training cohort and overall survival analyses. NMPR, neutrophil–mean-platelet-volume–platelet ratio.

As shown in Figure 1, the optimal cutoff values of NMPR were estimated by X-tile software which divided the studied population into a high-NMPR group (NMPR >16.62) and low-NMPR group (NMPR ≤ 16.62). The associations between NMPR and clinicopathologic parameters are summarized in Table 1. In the training cohort, there were 155 (56.0%) patients in the low-NMPR group and 122 (44.0%) patients in the high-NMPR group. The NMPR was found to be significantly associated with tumor length (*p* = 0.004) and CRP level (*p* = 0.028), but marginally associated with T stage (*p* = 0.055), while there was no significant association of NMPR with other clinicopathologic parameters. In the external validation cohort, there were 60 (59.4%) patients in the low-NMPR group and 41 (40.6%) patients in the high-NMPR group with the same cutoff value to the training cohort. The NMPR was found to be significantly associated with tumor location (*p* = 0.042), tumor length (*p* = 0.013) and T stage (*p* = 0.002), but not associated with other clinicopathologic parameters. Collectively, the results from the two cohorts both supported a significant association of NMPR with tumor length and T stage.

### 3.3. Associations between NMPR and Prognosis in Patients with Resectable ESCC

**Figure 2:**
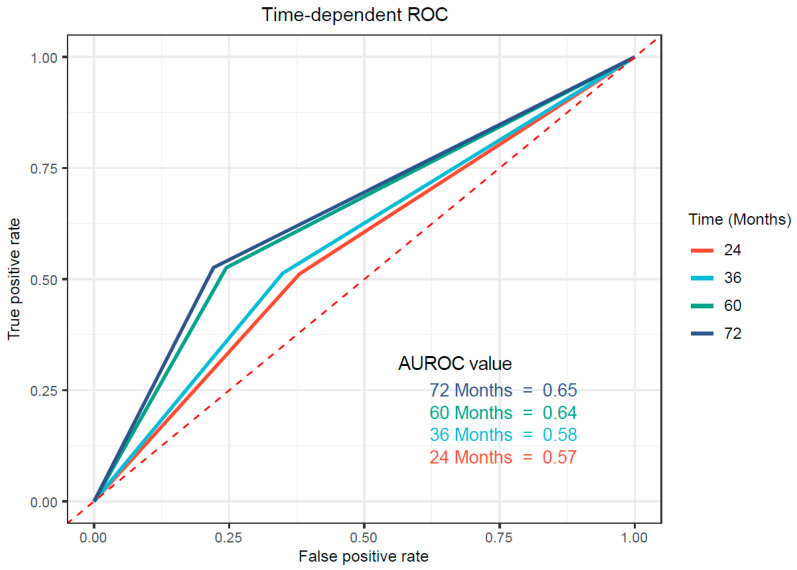
Predictive ability of NMPR in resectable esophageal squamous cell carcinoma by ROC curves in 24 months, 36 months, 60 months and 72 months in the training cohort. NMPR, neutrophil–mean-platelet-volume–platelet ratio.

**Figure 3:**
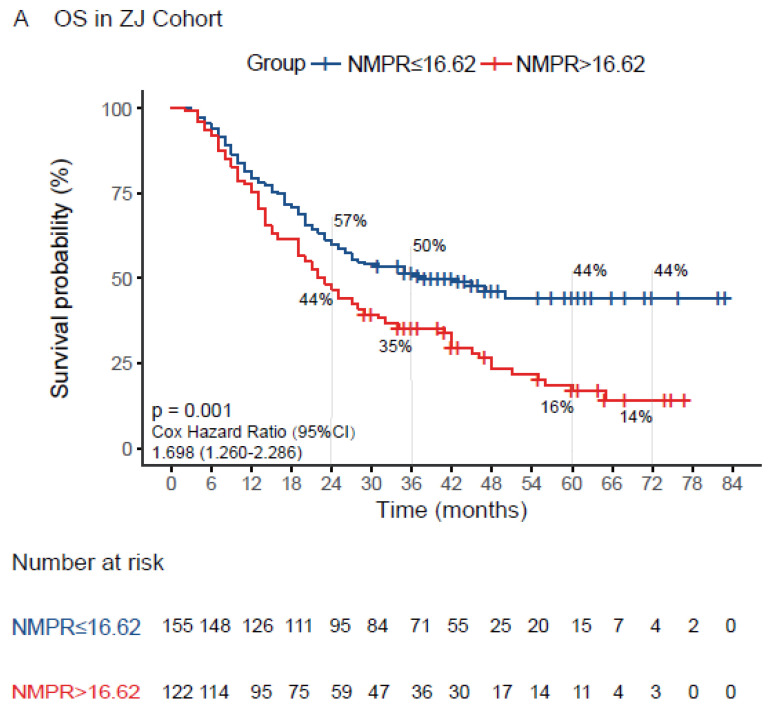

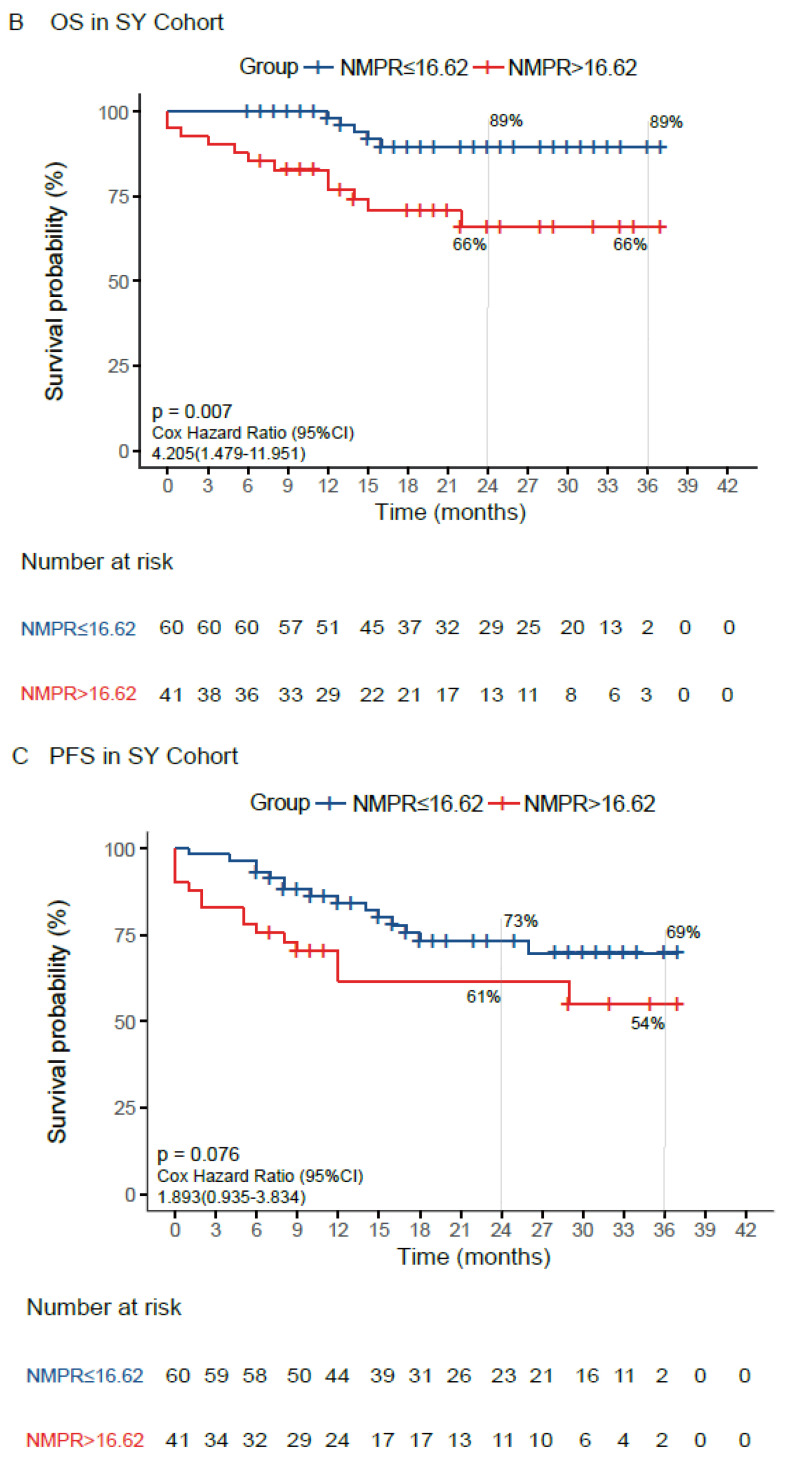
Kaplan–Meier curves for PFS or OS according to NMPR in the training cohort (**A**) and the external validation cohort (**B**,**C**). PFS, progression-free survival; OS, overall survival; NMPR, neutrophil–mean-platelet-volume–platelet ratio.

Firstly, the ROC curve was used to evaluate the ability of NMPR with established cutoff value to predict overall survival in the training cohort. As shown in Figure 2, the AUC value was 0.57, 0.58, 0.64 and 0.65 at 24 months, 36 months, 60 months and 72 months, respectively, which suggested a possibility that the NMPR was more able to predict the long-term survival outcome in resectable ESCC. Additionally, the Kaplan–Meier cumulative survival curve is plotted as Figure 3A with a median OS of 22 months (95% CI:17.7–26.3) in the high-NMPR group and a median OS of 38 months (95% CI:22.2–53.8) in the low-NMPR group. Correspondingly, the 2-, 3-, 5-, and 6-year OS rate were 44%, 35%, 16% and 14% in the high-NMPR group and 57%, 50%, 44% and 44% in the low-NMPR group, respectively. Then, univariate Cox regression analysis revealed that high NMPR (*p* = 0.001), tumor length (*p* = 0.002), vessel invasion (*p* = 0.004), poor tumor differentiation (*p* = 0.023), advanced T stage (*p* < 0.001), advanced N stage (*p* < 0.001), high CEA (*p* = 0.009) and high CRP (*p* < 0.001) were significantly associated with poor OS (Table 2). The multivariate Cox regression analysis involving significant factors indicated by the univariate Cox regression analysis demonstrated that high NMPR (*p* = 0.04) along with high CRP (*p* < 0.001), poor tumor differentiation (*p* = 0.008), advanced T stage (*p* = 0.006), advanced N stage (*p* < 0.001) and high CEA (*p* = 0.007) might be independent predictors for unfavorable OS (Table 2). Only OS was included in the analysis due to the lack of PFS data in the original dataset.

### 3.4. Validation of the Prognostic Value of NMPR in an External Cohort

The prognostic value of NMPR suggested by the training cohort was further validated with the same cutoff value in an external cohort. Likewise, the ROC curve was used to evaluate the ability of NMPR with established cutoff value to predict overall survival in the validation cohort. As shown in Figure S1, the AUC value was 0.68 and 0.53 at 24 months and 36 months, respectively. The AUC value was not determined for 60 months and 72 months due to a shorter follow-up period. For survival analysis, as shown in Figure 3B, the median OS and PFS were both not reached in the high-NMPR group and low-NMPR group. The 2- and 3-year OS rate were both 66% in high-NMPR group and were both 89% in low-NMPR group, respectively. Likewise, the 2- and 3-year PFS rate were 61% and 54% in the high-NMPR group and 73% and 69% in the low-NMPR group, respectively (Figure 3C). Similarly, the Cox regression analysis was performed to verify the prognostic value of NMPR. Notably, the CRP and CEA levels were not included in the Cox regression analysis due to the lack of relevant information in the validation cohort. As a result, the univariate Cox regression analysis revealed that high NMPR (*p* = 0.007), tumor length (*p* = 0.034), advanced T stage (*p* = 0.013) and advanced N stage (*p* = 0.019) were significantly associated with poor OS (Table 3). The multivariate Cox regression analysis revealed that only high NMPR (*p* = 0.049), and no other parameters, was independently associated with poor OS. Similarly, the univariate Cox regression analysis regarding PFS showed that the vessel invasion (*p* < 0.001), tumor length (*p* = 0.012), advanced T stage (*p* = 0.001) and advanced N stage (*p* < 0.001) were significantly associated with shorter PFS, while high NMPR (*p* = 0.076) was marginally associated with shorter PFS (Table 4). Correspondingly, the multivariate Cox regression analysis qualified that vessel invasion (*p* = 0.025) might independently predict PFS, although advanced T stage (*p* = 0.072) was marginally associated with shorter PFS (Table 4). Collectively, the results verified that high NMPR was a potential independent predictor of poor OS.

### 3.5. Development of NMPR-Based Nomogram for OS

**Figure 4:**
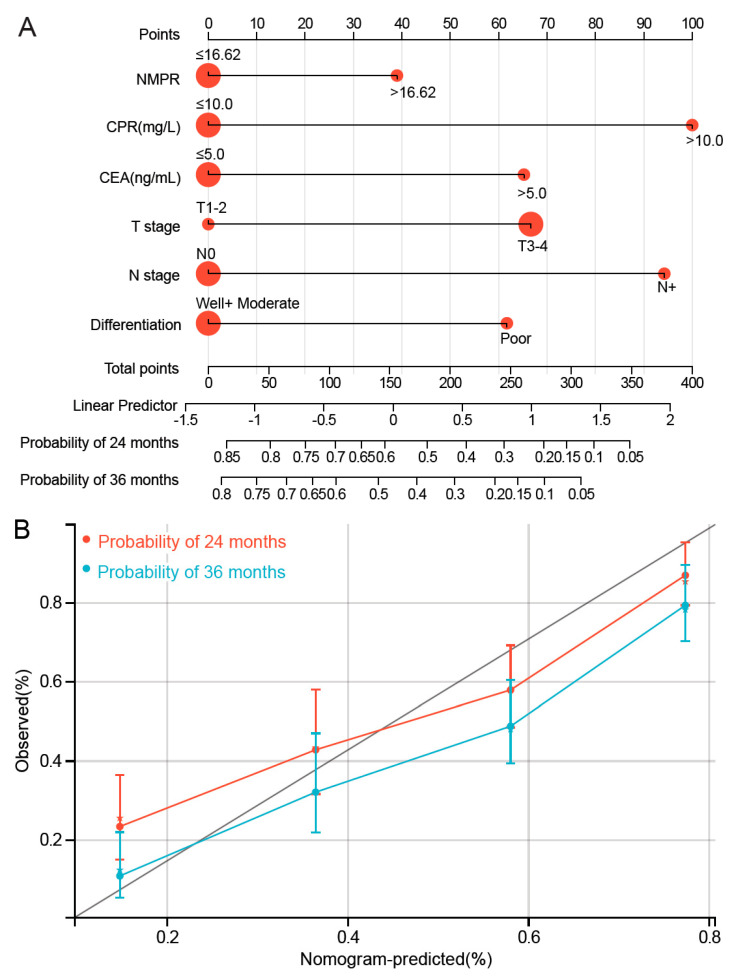
The nomogram (**A**) and calibration plots (**B**) for OS prediction in the training cohort. OS, overall survival; NMPR, neutrophil–mean-platelet-volume–platelet ratio; CRP, C-reaction protein; CEA, carcinoembryonic antigen.

In light of the limited sample size and lack of some information, the nomogram was only developed in the training cohort. The independent predictors including NMPR, CRP, CEA, T stage, N stage and tumor differentiation were used to establish nomogram (Figure 4A). The evaluation revealed that the predictive accuracy of the nomogram is acceptable with a C-index of 0.71 (95% CI; 0.676–0.748). The calibration plots of the nomograms also showed a good coherence between the predictions and actual values in the probability of 2- and 3-year OS (Figure 4B).

## 4. Discussion

For decades, the close link between cancer and inflammation has been widely accepted. An inflammatory tumor environment has proven detrimental by promoting the proliferation and survival of malignant cells as well as angiogenesis and metastasis in the development of ESCC [19]. Testing leukocytes obtained from peripheral blood is an inexpensive, highly reliable, and reproducible method to examine the status of systemic inflammatory responses, by which numerous valuable indicators such as NLR, PLR and RDW have been confirmed as prognostic, even treatment-guided, in a variety of cancers including ESCC [3,20,21,22]. In the present study, the first of its kind to the best of our knowledge, we investigated and validated the prognostic value of NMPR, demonstrating an adverse impact of high NMPR on OS in patients with resectable ESCC. Moreover, we developed a nomogram incorporating NMPR, T stage, N stage, tumor differentiation, CEA and CRP to predict the OS with reasonable discriminations and calibrations.

Accumulating evidence shows that the systemic inflammatory response is associated with clinical outcome in multiple types of cancers [23]. Among the peripheral blood cells, neutrophils and platelets are important elements involved in systemic inflammation and the main contributors affecting patient outcomes. Mechanically, the neutrophil population increased under inflammatory conditions, such as in a tumor-bearing body, secreting a large amount of nitric oxide, arginase, cytokines and reactive oxygen species, resulting in disorders of T cell activation and stimulating the production of vascular endothelial growth factor (VEGF) to accelerate tumor neovascularization [24,25,26]. Activated platelets were also a critical source of growth factors and cytokines, especially transforming growth factor β and VEGF to support multiple malignant biological behaviors of cancer cells [3], and platelet count was often found increased in patients with solid tumors [27,28]. Similarly, another platelet indices, MPV, which was usually recognized as a hallmark for platelet activation, was used to reflect the function status of platelets under inflammatory conditions, and MPV/PC was classically used as a marker of platelet activation in peripheral blood [29]. Thus, clinically, elevated levels of neutrophil, PC or MPV were useful indicators of systemic inflammation in cancer patients, which were also linked to a poor prognosis in a variety of cancers including ESCC.

Recently, studies showed that platelet activation triggers platelet–neutrophil interaction and modulates the immune system, leading to the progression of inflammation condition in several inflammatory diseases [30,31,32]. Similar interaction between platelets and neutrophils was also found in cancer development and progression. The direct and indirect interplay between platelet and neutrophil was able to regulate several aspects of tumor-associated pathology and influence the tumor immune microenvironment in cancer at various stages [33]. Further mechanistic investigation demonstrated that platelets might augment rolling and firm neutrophil adhesion via the crosstalk between platelets and neutrophils initiated by the platelet surface marker P-selectin (CD62P) and neutrophil surface marker P-selectin-binding glycoprotein 1 (PSGL-1) [34,35]. Subsequently, platelet–neutrophil interaction fostered mutual activation and neutrophil extracellular traps and triggered the release of platelet granule contents and the generation of lipid mediators, which affected the functions of various effector immune cells including T-helper, CD8+ T and natural killer cells in the tumor environment via various cytokines, chemokines and prostaglandins signaling such as Toll-like receptors, interleukin-1 (IL-1), IL-17, CCL2, IL-8, PD-L1, IFN-γ, and TNF-α, etc., facilitating immune evasion of cancer cells and tumor development and dissemination [33]. Therefore, the measurement of combined neutrophil and platelet indices values may reflect the pro-tumor inflammatory status more accurately in cancer patients.

In the present study, we developed a novel inflammation-based NMPR score comprised of neutrophil and MPV to Log (PC) ratio, of which the prognostic value has yet to be examined in cancer patients. Our study investigated the effect of preoperative NMPR level on the survival of ESCC patients, demonstrating a significant association of high NMPR with shorter OS. Moreover, the multivariate Cox regression analysis suggested that NMPR might be an independent prognostic indicator for ESCC, which was further validated in an external cohort to add the reliability of the results. However, a borderline but not significant association of NMPR with PFS was revealed (*p* = 0.07), which may be due to the limited sample size in the validation cohort or heterogeneity in neoadjuvant therapy or surgery schedule. Indeed, in several previous studies, a part of NMPR, MPV/PC ratio has been reported to predict the clinical outcome of resectable, locally advanced and apatinib-treated advanced ESCC, respectively [11,17,36]. However, the NMPR seemed to improve the prediction efficiency for long survival with higher AUROC value compared to MPV/PC in the previous study (0.64 vs. 0.60) [17], suggesting the superior value of NMPR in survival prediction of resectable ESCC. Similarly, the prognostic value of another method of combined neutrophil and platelet indices expressed as neutrophil–platelet score (NPS, neutrophils ≤ 7.5 × 10^9^/L and platelets ≤ 400 × 10^9^/L defined as score of 0; neutrophils ≥ 7.5 × 10^9^/L or platelets ≥ 400 x10^9^/L defined as score of 1; neutrophils > 7.5 × 10^9^/L or platelets > 400 × 10^9^/L defined as score of 2) was investigated in locally advanced ESCC treated with chemoradiotherapy as well as other cancers [14,16]. Consistent with our findings, high NPS was found to be associated with poor survival in a variety of common cancers including gastroesophageal cancer, implying the high value of combined neutrophil and platelet indices in esophageal cancer patients. However, the main difference between NMPR and NPS was whether the MPV was included or not. As mentioned previously, the systemic pro-tumor inflammatory status may be reflected by neutrophils, platelets and their interaction, and the activation of platelets is a key step in the release of a large amount of inflammatory factors and the initiation of inflammation process to favor the tumor, which is likely indicated by MPV. Biologically, it is, therefore, more plausible to include MPV when combining neutrophils and platelets for the evaluation of systemic inflammatory conditions in cancer patients. Additionally, the relationship between NMPR and clinicopathologic parameters was also investigated in the study. We found that high NMPR was significantly associated with several unfavorable factors including longer tumor length, advanced T stage and high CRP level, supporting a potential association of NMPR with increased inflammation induced by cancer progression. Furthermore, with the ZJ test cohort, this study identified several other prognostic factors including tumor differentiation, T stage, N stage, CEA and CRP, which were well-established in previous studies regarding esophageal cancer [2,17,37,38,39]. However, their prognostic value was not validated in the external RM validation cohort, which may be explained by several points: limited sample size, short follow-up, heterogeneity of baseline characteristics between test and validation cohorts, missing data such as CEA and CRP in RM cohort, difference in adjuvant therapy and late-line treatment. Using the ZJ cohort with a larger sample size, we further constructed a nomogram with clinical parameters of T stage, N stage, tumor differentiation, and preoperative NMPR, CRP and CEA levels. The nomogram was evaluated and considered reliable with an acceptable predictive accuracy by exhibiting an acceptable calibration curve, based on looking at the nomograph prediction probability versus actual probability and presenting a concordance index (c-index) of 0.71. Our result, however, needed further validation.

It is noteworthy that our study is the first attempt to evaluate the prognosis significance of inflammation-based index NMPR in patients with ESCC. The NMPR is simple and comprised of components of a blood test with low cost, which is of great value for the patients in developing countries and low-income territories. Still, there are several potential limitations in the current study. First, the retrospective study has an inherent selection bias. Although the prognostic value of NMPR was revealed, the multivariate Cox regression analysis in the external validation cohort was not adjusted by several important factors, such as CEA and CRP levels, due to the missing data which were not routinely examined preoperatively. Likewise, data asymmetry of the baseline characteristics between the high and low NMPR group and between the test and validation cohorts might affect the result to some extent as well, which was indicated by the significant difference in survival outcome between the test and validation cohort (Figure 3). Further, considering that blood parameters were dynamic, blood samples were not obtained at an accordant timepoint and without repeated test, which might introduce an irreconcilable bias and negate the utility of the test. Then, different equipment among different medical institutions may result in inconsistency in the results of blood parameter tests, which may affect the applicability of our findings. Lastly, the nomogram was constructed using several significant prognostic parameters in the test cohort, which was not validated by an external cohort. The reliability and generalization are therefore limited. Finally, there might be insufficient power to validate the significance of NMPR with a short follow-up period in the cohort with relatively small sample size, considering the lack of a significant association of NMPR with PFS. Our findings should be interpreted with caution in light of the defects mentioned above.

## 5. Conclusions

In conclusion, the present study revealed that preoperative elevated NMPR was associated with unfavorable clinicopathologic features in resectable ESCC patients. The results of our study emphasize the importance of combined neutrophil and platelet indices by demonstrating that preoperative elevated NMPR was independently associated with poor survival outcome in patients with resectable ESCC. Due to its ease of accessibility and low cost, large-scale prospective validation studies are warranted to confirm these results to promote its clinical application.

## Figures and Tables

**Table 1:** Baseline characteristics of the training cohort (ZJ Cohort) and external validation cohort (SY cohort) according to NMPR group.

Characteristics	Total	NMPR of ZJ Cohort		*p*-Value	Total	NMPR of SY Cohort		*p*-Value	*p*-Value
	(N = 277)	≤16.62 (n = 155)	>16.62 (n = 122)		(N = 101)	≤16.62 (n = 60)	>16.62 (n = 41)		
Age (years)				0.886				0.299	0.008
≤60	158 (57%)	89 (56%)	69 (44%)		42 (42%)	23 (55%)	19 (45%)		
>60	119 (43%)	66 (55%)	53 (45%)		59 (58%)	37 (63%)	22 (37%)		
Gender				0.126				0.631	0.899
Female	37 (13%)	25 (68%)	12 (32%)		14 (14%)	9 (64%)	5 (36%)		
Male	240 (87%)	130 (54%)	110 (46%)		87 (86%)	51 (59%)	36 (41%)		
Tumor length (cm)				0.004				0.013	0.001
≤3.0	74 (27%)	52 (70%)	22 (30%)		46 (46%)	33 (72%)	13 (28%)		
>3.0	203 (73%)	103 (51%)	100 (49%)		55 (54%)	27 (49%)	28 (51%)		
Tumor location				0.488				0.042	<0.001
Upper + Middle	145 (52%)	84 (58%)	61 (42%)		22 (22%)	17 (77%)	5 (23%)		
Lower	132 (48%)	71 (54%)	61 (46%)		79 (78%)	43 (54%)	36 (46%)		
Vessel invasion				0.474				0.719	<0.001
Negative	232 (84%)	132 (57%)	100 (43%)		58 (57%)	33 (57%)	25 (43%)		
Positive	45 (16%)	23 (51%)	22 (49%)		43 (43%)	27 (63%)	16 (37%)		
Differentiation				0.326				0.152	0.364
Well+ Moderate	223 (81%)	128 (57%)	95 (43%)		77 (76%)	48 (62%)	29 (38%)		
Poor	54 (19%)	27 (50%)	27 (50%)		24 (24%)	12 (50%)	12 (50%)		
T stage				0.055				0.002	0.491
T1 + T2	99 (36%)	63 (64%)	36 (36%)		40 (40%)	31 (76%)	9 (24%)		
T3 + T4	178 (64%)	92 (52%)	86 (48%)		61 (60%)	29 (48%)	32 (52%)		
N stage				0.616				0.338	<0.001
N0	150 (54%)	86 (57%)	64 (43%)		77 (76%)	47 (61%)	30 (39%)		
N+	127 (46%)	69 (54%)	58 (46%)		24 (24%)	13 (54%)	11 (46%)		
TNM stage				0.337				0.312	0.004
I + II	161 (58%)	94 (58%)	67 (42%)		75 (74%)	46 (61%)	29 (39%)		
III	116 (42%)	61 (53%)	55 (47%)		26 (26%)	14 (54%)	12 (46%)		
CEA (ng/mL)				0.926					
≤5.0	239 (86%)	134 (56%)	105 (44%)						
>5.0	38 (14%)	21 (55%)	17 (45%)						
CRP (mg/L)				0.028					
≤10.0	198 (71%)	119 (60%)	79 (40%)						
>10.0	79 (29%)	36 (46%)	43 (54%)						

NMPR, neutrophil–mean-platelet-volume–platelet ratio; TNM, tumor node metastasis; CEA, carcinoembryonic antigen; CRP, C-reactive protein.

**Table 2:** Univariate and multivariate Cox regression analyses for overall survival in the training cohort (ZJ Cohort).

Variable	Univariate		*p*-Value	Multivariate		*p*-Value
	HR	95% CI		HR	95% CI	
Age (≤60 y/>60 y)	1.088	0.806–1.468	0.583			
Gender (Female/Male)	1.392	0.854–2.268	0.185			
Tumor length (≤3 cm/>3 cm)	1.767	1.224–2.553	0.002	1.106	0.734–1.667	0.630
CRP (≤10 ng/mL/>10 ng/mL)	2.258	1.652–3.083	<0.001	2.383	1.718–3.307	<0.001
Tumor location (Upper + Middle/Lower)	1.168	0.868–1.573	0.305			
Vessel invasion (Negative/Positive)	1.725	1.194–2.492	0.004	0.986	0.669–1.453	0.942
Differentiation (Well + Moderate/Poor)	1.515	1.060–2.165	0.023	1.674	1.143–2.452	0.008
T stage (T1-2/T3-4)	2.170	1.541–3.057	<0.001	1.718	1.168–2.528	0.006
N stage (N0/N+)	2.626	1.932–3.569	<0.001	2.257	1.632–3.123	<0.001
CEA (≤5 mg/L/>5 mg/L)	1.707	1.114–2.546	0.009	1.752	1.167–2.630	0.007
Adjuvant therapy (No/Yes)	1.291	0.944–1.766	0.110			
NMPR (≤16.62/>16.62)	1.698	1.260–2.286	0.001	1.380	1.014–1.878	0.040

NMPR, neutrophil–mean-platelet-volume–platelet ratio; HR, hazard ratio; CI, confidence interval; CRP, C-reactive protein; CEA, carcinoembryonic antigen. The former of the group in every variable was set as reference in Cox regression analyses.

**Table 3:** Univariate and multivariate Cox regression analyses for overall survival in the external validation cohort (SY Cohort).

Variable	Univariate		*p*-Value	Multivariate		*p*-Value
	HR	95% CI		HR	95% CI	
Age (≤60 y/>60 y)	1.869	0.657–5.311	0.241			
Gender (Female/Male)	2.591	0.344–19.545	0.356			
Tumor length (≤3 cm/>3 cm)	3.362	1.092–10.345	0.034	1.148	0.303–4.350	0.840
Tumor location (Upper + Middle/Lower)	2.313	0.528–10.125	0.266			
Vessel invasion (Negative/Positive)	2.417	0.894–6.537	0.082			
Differentiation (Well + Moderate/Poor)	1.009	0.329–3.097	0.987			
T stage (T1-2/T3-4)	6.490	1.477–28.510	0.013	3.226	0.588–18.141	0.176
N stage (N0/N+)	3.141	1.211–8.151	0.019	2.165	0.785–5.972	0.136
NMPR (≤16.62/>16.62)	4.205	1.479–11.951	0.007	3.029	1.007–9.112	0.049

NMPR, neutrophil–mean-platelet-volume–platelet ratio; HR, hazard ratio; CI, confidence interval; The former of the group in every variable was set as reference in Cox regression analyses.

**Table 4:** Univariate and multivariate Cox regression analyses for progress-free survival in the external validation cohort (SY Cohort).

Variable	Univariate		*p*-Value	Multivariate		*p*-Value
	HR	95% CI		HR	95% CI	
Age (≤60 y/>60 y)	1.701	0.800–3.616	0.168			
Gender (Female/Male)	5.510	0.751–40.433	0.093			
Tumor length (≤3 cm/>3 cm)	2.748	1.254–6.019	0.012	1.406	0.592–3.337	0.440
Tumor location (Upper + Middle/Lower)	1.048	0.451–2.434	0.913			
Vessel invasion (Negative/Positive)	4.689	2.094–10.499	<0.001	2.788	1.134–6.852	0.025
Differentiation (Well + Moderate/Poor)	1.738	0.814–3.709	0.153			
T stage (T1-2/T3-4)	5.164	1.959–13.612	0.001	2.709	0.915–8.023	0.072
N stage (N0/N+)	3.775	1.851–7.702	<0.001	1.637	0.915–8.023	0.224
NMPR (≤16.62/>16.62)	1.893	0.935–3.834	0.076			

NMPR, neutrophil–mean-platelet-volume–platelet ratio; HR, hazard ratio; CI, confidence interval; The former of the group in every variable was set as reference in Cox regression analyses.

## Data Availability

The dataset of ZJ cohort was public and derived from a previous publication by Feng et al. (https://peerj.com/articles/7246/#supplemental-information, accessed on 27 June 2022). The dataset in RM cohort was available from the corresponding author on reasonable request.

## Supplementary Materials

The following are available online at https://www.mdpi.com/article/10.3390/medicina58121808/s1, Figure S1: Predictive ability of NMPR in resectable esophageal squamous cell carcinoma by ROC curves in 24 months and 36 months in the validation cohort. NMPR, neutrophil mean platelet volume platelet ratio.
