# Supplementary material for: Elevated Preoperative NMPR Predicts an Unfavorable Chance of Survival in Resectable Esophageal Squamous Cell Carcinoma
Source: Medicina (Kaunas). 2022 Dec 8;58(12):1808. doi: 10.3390/medicina58121808 (PMC9788475; doi:10.3390/medicina58121808)
Supplement: Supplementary file 1 [file medicina-58-01808-s001.zip › medicina-2015687-supplementary.pdf]

Supplementary Materials

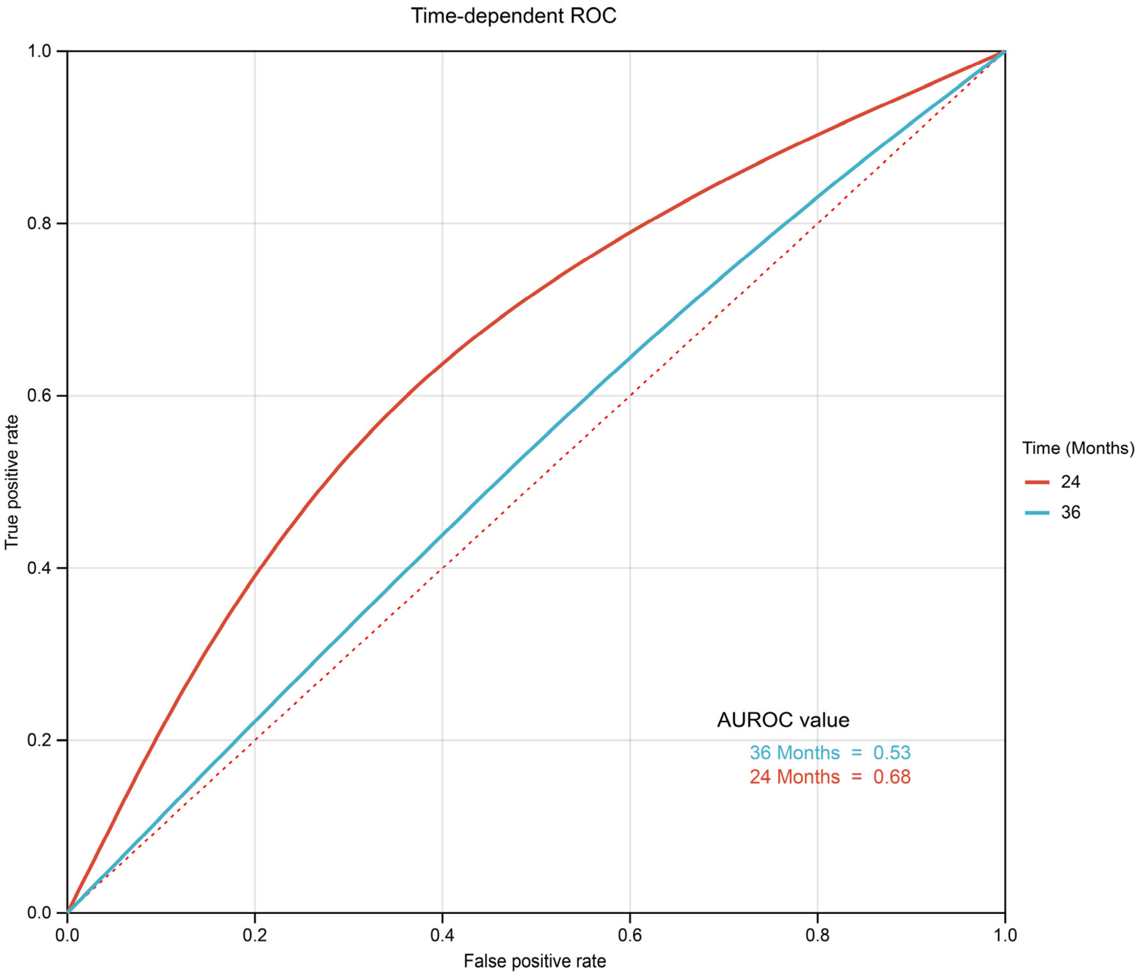

Figure S1. Predictive ability of NMPR in resectable esophageal squamous cell carcinoma by ROC curves in 24 months and 36 months in the validation cohort. NMPR, neutrophil–mean-platelet-volume–platelet ratio.
